# Quantitative Disk and Metabolic Bone PET‐MRI for Understanding Disk‐Endplate Crosstalk in Degeneration of the Lumbar Spine

**DOI:** 10.1002/jsp2.70188

**Published:** 2026-06-08

**Authors:** Virginie Kreutzinger, Katharina Ziegeler, Misung Han, Cynthia T. Chin, Rupsa Bhattacharjee, Isabelle Remick, Emma Bahroos, Sharmila Majumdar

**Affiliations:** ^1^ Department of Radiology and Biomedical Imaging University of California San Francisco San Francisco California USA; ^2^ Department of Neuroradiology TUM Klinikum, Technical University of Munich Munich Germany; ^3^ Department of Medical Science and Technology Indian Institute of Technology Madras Chennai India

**Keywords:** Na[^18^F]F‐PET MRI, quantitative MRI, T1rho disc, vertebral endplate degeneration

## Abstract

**Objectives:**

The aim of this investigation was to explore the association between disk degeneration, quantified by Pfirrmann grades and T1rho as a marker of hydration, and bone remodeling in the vertebral endplate quantified by Na[^18^F]F uptake on PET.

**Methods:**

Subjects for this exploratory, post hoc analysis were selected from a prospective observational study on low back pain. Subjects received simultaneous Na[^18^F]F‐PET and spinal MRI (including T1rho mapping) in a hybrid scanner system. Tracer uptake (SUV_max_) was quantified per endplate, and quantitative disk metrics were measured for annulus fibrosus and nucleus pulposus separately. Disk degeneration (Pfirrman grade) and endplate lesions (Modic changes) were assessed by a radiologist. Linear mixed models were used to investigate the association between quantitative disk and metabolic endplate metrics, adjusting for age, sex, body mass index, as well as Pfirrmann grade and Modic changes.

**Results:**

Eighty‐six endplates from 9 patients (6 women) with median age 58 years (IQR: 53–72) were included. We found a positive association between annular T1rho and endplate Na[^18^F]F uptake (*β* = 0.088; 95% CI 0.018–0.159). The association was amplified by the presence of Modic changes (interaction *p* = 0.001).

**Conclusion:**

Our findings indicate that bone remodeling of vertebral endplates is not associated with disk dehydration, and thus more likely a result of biological disk‐endplate crosstalk than a sign of increased mechanical load.

## 1 Introduction

Chronic low back pain (CLBP) is among the leading causes of disability and incurs substantial and rising direct and indirect healthcare costs in the United States and worldwide [1, 2]. It can be caused by a heterogeneous group of conditions, most of which can be detected on magnetic resonance imaging (MRI), which constitutes a central pillar in the diagnostic work‐up of CLBP. A tissue that is well‐accepted to be involved in pain generation, especially in axial CLBP is the disco‐vertebral complex, that is, the interface of the vertebral disk and endplate [3]. One of the earliest descriptions of the association between vertebral endplate changes visualized on MRI and CLBP was supplied by Modic et al. [4]. These changes, divided into Type 1 (edematous), Type 2 (fatty), and Type 3 (sclerotic) lesions, are well‐established findings in degenerative disk disease, and the association of Type 1 changes and LBP have been shown to be robust in larger meta‐analyzes [5, 6]. While data from conventional MRI is abundant, less is known about the histology and pathobiology of these lesions, especially their early stages and progenitor lesions [7]. Existing smaller studies using surgical tissues or biopsies have demonstrated fibrovascular granulation tissue at the disk/endplate junction and altered rates of bone turnover in these lesions [8, 9]. As studies using tissue samples are highly invasive and typically performed in patients undergoing surgery, these types of analyzes are typically limited to late‐stage lesions. Less invasive techniques to study endplate‐disk crosstalk, especially in earlier stages, could be found in quantitative imaging techniques. One such marker, that has gained traction in disk health research is T1rho, which is correlated with discal pressure (i.e., hydration) and glycosaminoglycan content [10]. Due to its quantitative nature it can pick up subtle early changes in disk composition, that may not be discernible by human assessors and has shown good sensitivity to disk degeneration [11]. An emerging tool for the noninvasive quantification of bone remodeling is positron emission tomography (PET)‐MRI, using Na[^18^F]F as the injected radiotracer [12]. In the context of degenerative spine disease, it has been used to detect facet joint related pain [13]. Na[^18^F]F PET‐MRI furthermore shows promise in elucidating bone‐cartilage crosstalk in the context osteoarthritis [14, 15] by visualizing areas of increased bone turnover. To date, no studies exist that specifically investigate the association between quantitative imaging biomarkers of disk health and bone remodeling at the vertebral endplate.

The aim of this study was therefore to investigate the association of quantitative disk health and metabolic bone markers in LBP patients. Our hypothesis was that bone remodeling in the vertebral endplate is negatively associated with disk hydration, as decreased cushioning of desiccated disks may lead to microfractures and subsequent build‐up of bony matrix, that is, sclerosis.

## 2 Methods

### 2.1 Study Participants

Patients for this ancillary analysis were drawn from the ongoing longitudinal observational technology development study, conducted at our institution as part of the National Institutes of Health (NIH) Back Pain Consortium (BACPAC) [16]. This study recruited individuals with low back pain (minimum duration 3 months) via referring physicians. Exclusion criteria for the parent study were age < 18 years, inability to provide written informed consent, pregnancy or current breast‐feeding, recent (< 12 months) history of fracture, tumor or operation of the spine, rheumatic disease (e.g., axial spondyloarthritis), active malignancy, contraindication for MR imaging or injection of radiotracer. Of the overall 87 study subjects we excluded individuals that received no PET‐imaging (*n* = 65) or PET‐imaging with nonbone specific radiotracer than [^18^F]FDG (*n* = 11), as well as individuals with so advanced multilevel disk degeneration, that quantitative disk assessment was not feasible (*n* = 2). All participants provided written informed consent prior to study activities, and Internal review Board (IRB) approval was granted before commencement of the study (IRB 19‐29744).

### 2.2 Image Acquisition

All study subjects underwent imaging in a 3.0T whole‐body hybrid PET/MRI scanner (GE Healthcare, Waukesha, Wisconsin, USA). The clinical spinal MRI protocol included sagittal T1‐weighted 2D fast spin‐echo (FSE) and sagittal T2‐weighted FSE with and without fat suppression, axial T1‐weighted FSE, and axial T2‐weighted FSE sequences; technical details have been described in previous work of our group [17]. Magnetization‐prepared angle‐modulated partitioned k‐space spoiled gradient echo snapshots (MAPSS) [18, 19] were used for T1rho and T2 quantification. Imaging parameters included a field‐of‐view (FOV) of 20 × 20 cm^2^, 256 × 128 matrix size, 6 mm slice thickness, 14 slices, and ±62.5 kHz readout bandwidth, and chemical‐selective fat suppression was incorporated prior to magnetization preparation. For T1rho quantification, spin‐lock times of 0, 10, 40, 80 ms and a spin lock frequency of 300 Hz were used while for T2 quantification, TE times of 0, 19.6, 39.3, and 78.6 were used. After image reconstruction, T1r and T2 were estimated using the four echo signals through exponential curve fitting employing the Levenberg–Marquardt method [20]. For PET, the administered dose of Na[^18^F]F averaged at 2.98 (± 0.09) mCi; intravenous tracer injection was followed by a 45 min seated rest period prior to image acquisition. The PET signal acquisition was performed for the first 20 min of the Na[^18^F]F PET‐MRI scan and all signals acquired during the acquisition window were used to generate a single static PET image set. The standardized uptake value (SUV) images were derived from the source static PET images reconstructed with the ordered subset expectation maximization (OSEM [21]). Examination times were held constant (between 10 am and noon) across subjects to account for possible diurnal changes in disk hydration.

### 2.3 Image Analysis

Both quantitative and qualitative/semiquantitative image assessments were per‐formed by a board‐certified radiologist (V.K., 7 years of experience), using dedicated software (Visage, Version 7.1.18, Pro Medicus Ltd., Richmond, Australia).

**FIGURE 1:**
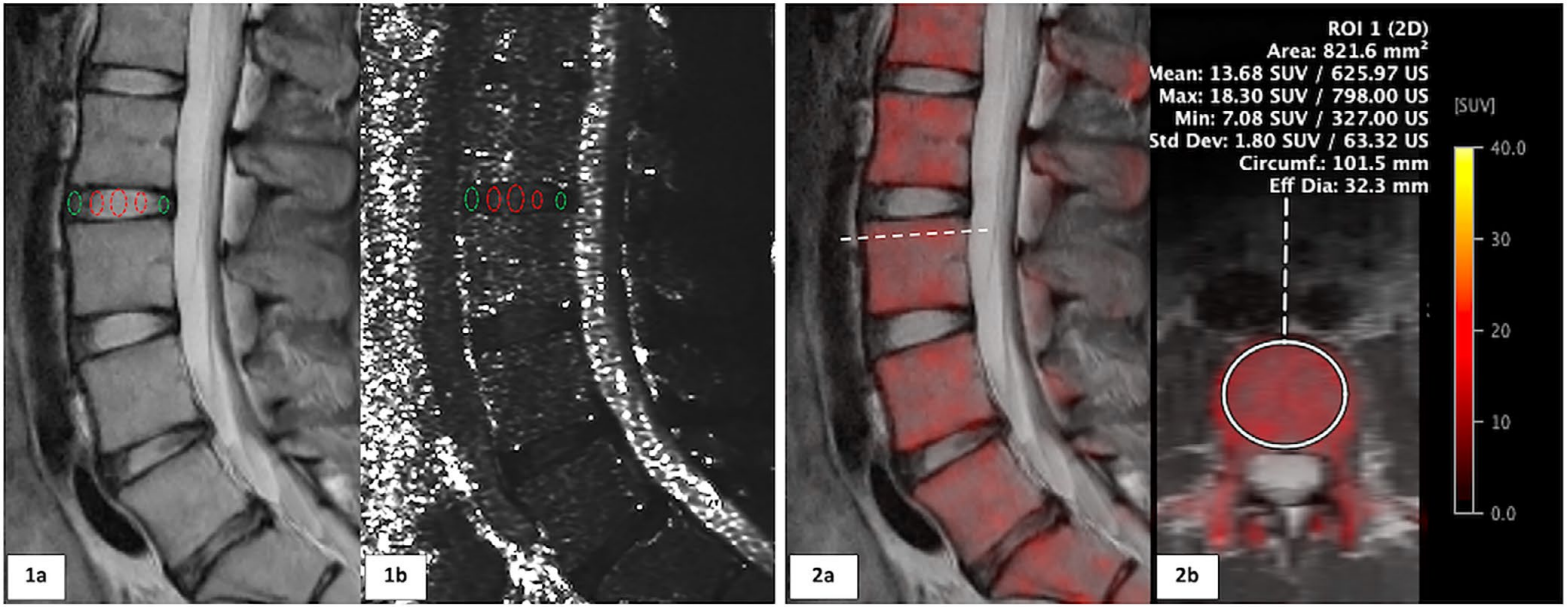
Quantitative data collection. Example images for collection of quantitative disk (1) and endplate (2) data in a 37 year‐old male subject. Images 1a (T2‐weighted spin‐echo image in sagittal plane) and 1b (T1rho map in sagittal plane) demonstrate region of interest (ROI) placement for annulus (green circles) and nucleus (red circles), which were placed in a central sagittal slice. Images 2a (Fusion image of PET‐map and T2‐weighted spin‐echo image in sagittal plane) and 2b (axial reconstruction parallel to the disk, indicated by dotted white line) demonstrate collection of quantitative endplate metric.

Regions of interest (ROI) were placed in predefined sample locations for the extraction of quantitative metrics. For Na[^18^F]F uptake quantification, elliptical ROIs were placed in the bone immediately adjacent to the endplate (range: inferior endplate L1 to superior endplate S1) on axial images and maximum standardized uptake value (SUV_max_) was noted. SUV_max_ was preferred over SUV_mean_ because bone remodeling in degenerative disk disease is spatially heterogeneous within the subchondral bone, and SUV_mean_ might dilute the peak metabolic signal by averaging across both active and quiescent regions within the ROI. For both T1rho and T2 values, ROIs were placed in a total of five sample regions within the disk (two annulus fibrosus, three nucleus pulposus) in a representative mid‐sagittal slice, and respective maximum values were noted. A visualization of ROI placements is provided in Figure 1. Individual disks with end‐stage degeneration, defined as loss of discernible disk tissue within the intervertebral space, were excluded from the analysis (*n* = 4). Reproducibility of our measurement technique was high with an intraclass correlation coefficient of 0.97 (95% CI 0.95, 0.98), as described in detail in previous work [22].

All qualitative assessments were performed in accordance with the recommendations of the BACPAC spine imaging group [23]. Degenerative lesions of the vertebral endplates were assessed qualitatively, according to the Modic classification [4]: 0 denotes no distinguishable lesion, 1 denotes an increase in fluid signal adjacent to the cartilage endplate (i.e., high signal intensity on T2w with concomitant low signal intensity on T1w images), 2 denotes fat metaplasia adjacent to the cartilage endplate (i.e., high signal intensity on both T2w and T1w images), and 3 denotes sclerosis adjacent to the cartilage endplate (i.e., low signal intensity on both T2w and T1w images). Cases of mixed Modic lesions (i.e., containing both fat and edema) were graded as Modic 1. Each endplate from the inferior endplate of L1 to the superior endplate of S1 received a separate grade. Degenerative disk changes were assessed using the Pfirrman grading system [24], which grades the loss of height and T2 signal on a five point scale, ranging from homogeneous with bright hyperintense signal and normal disk height (Grade I) to inhomogeneous hypointense signal, lack of differentiation between annulus fibrosus and nucleus pulposus and/or a collapsed disk space. Each disk from L1/2 to L5/S1 received a separate grade.

### 2.4 Statistical Analysis

As units of observation, individual endplates were chosen for this analysis. Descriptive statistics of the patient population were given as frequencies with percentages or median with interquartile range (IQR), due to small sample size with lack of normal distribution. To measure the association between endplate bone remodeling (SUV_max_ of Na[^18^F]F) and quantitative disk biomarkers (T1rho of annulus and nucleus, annulus‐to‐nucleus ratios), we used a cluster‐robust ordinary least‐squares (OLS) regression, adjusting for age (continuous), sex (binary), BMI (continuous), Pfirrmann grade (ordinal: 1–5), presence of annular fissure (binary), and any Modic change (binary), and including an interaction term for Modic changes as a secondary model. Variance inflation factors were all < 2.6, indicating absence of problematic multicollinearity with these models. Due to the exploratory nature of this study, we performed a post hoc power‐analysis (two‐sided *α* = 0.05), based on the observed within‐spine annulus T1rho slope (*β* = 0.088 SUV ms^−1^, SE = 0.030), and nine subject clusters yielded ≈73% power to detect this effect. Results with two‐sided *p*‐values < 0.05 were considered statistically significant. All analyzes were performed using Python Version 3.11.8.

## 3 Results

### 3.1 Study Participants

A total of nine subjects contributed 86 observations (paired endplate and disk metrics). Median patient age was 58 years (IQR: 53–72), the majority was female (67%; 6/9) and median BMI was 25.7 (IQR 25.4–27.4). Applying a conversion coefficient of 0.024 mSv/MBq [25], the mean calculated radiation exposure from Na[^18^F]F was 2.64 (±0.08) mSv.

### 3.2 Disk Changes

A compilation of both quantitative and qualitative disk metrics is provided in Table 1. We observed an expected slight decrease of mean T1rho and T2 values for both annulus and nucleus across a cranio‐caudal gradient, with lowest values at the L5‐S1 disk level. Annulus‐to‐nucleus ratios were highest at the L5‐S1 disk level but showed a less pronounced gradient. Degeneration assessed on the Pfirrman grade increased from higher to lower levels and exhibited a median Grade of 3 (IQR 2–4).

**TABLE 1:** Descriptive results for disc metrics per level.

Disc	T1rho [mean ms, SD]			T2 [mean ms, SD]			Pfirrmann grade [median, IQR]	Annulus fibrosus fissure [%, *n*]
	Annulus	Nucleus	A/N ratio	Annulus	Nucleus	A/N ratio		
L1–L2	52.9 (11.1)	67.8 (24.5)	0.82 (0.12)	49.0 (10.1)	64.8 (24.3)	0.80 (0.13)	2 (1.75–2.25)	0% (0/8)
L2–L3	50.2 (6.6)	68.4 (18.6)	0.77 (0.15)	51.2 (9.6)	65.9 (15.7)	0.80 (0.15)	3 (1–3)	22% (2/9)
L3–L4	53.9 (10.4)	69.5 (17.3)	0.80 (0.17)	50.8 (10.3)	69.9 (20.3)	0.76 (0.17)	3 (2–4)	44% (4/9)
L4–L5	49.6 (10.2)	62.0 (13.3)	0.81 (0.09)	45.6 (12.6)	58.2 (14.2)	0.79 (0.12)	3 (3–4)	56% (5/9)
L5–S1	43.1 (8.4)	51.0 (11.9)	0.86 (0.08)	38.8 (8.9)	47.4 (11.7)	0.83 (0.13)	4 (3–4)	75% (6/8)
Overall	50.1 (10.0)	63.9 (18.5)	0.81 (0.13)	47.2 (11.1)	61.5 (19.0)	0.79 (0.14)	3 (2–4)	40% (34/86)

Abbreviations: A/N ratio = annulus‐to‐nucleus ratio; IQR = interquartile range; SD = standard deviation.

**FIGURE 2:**
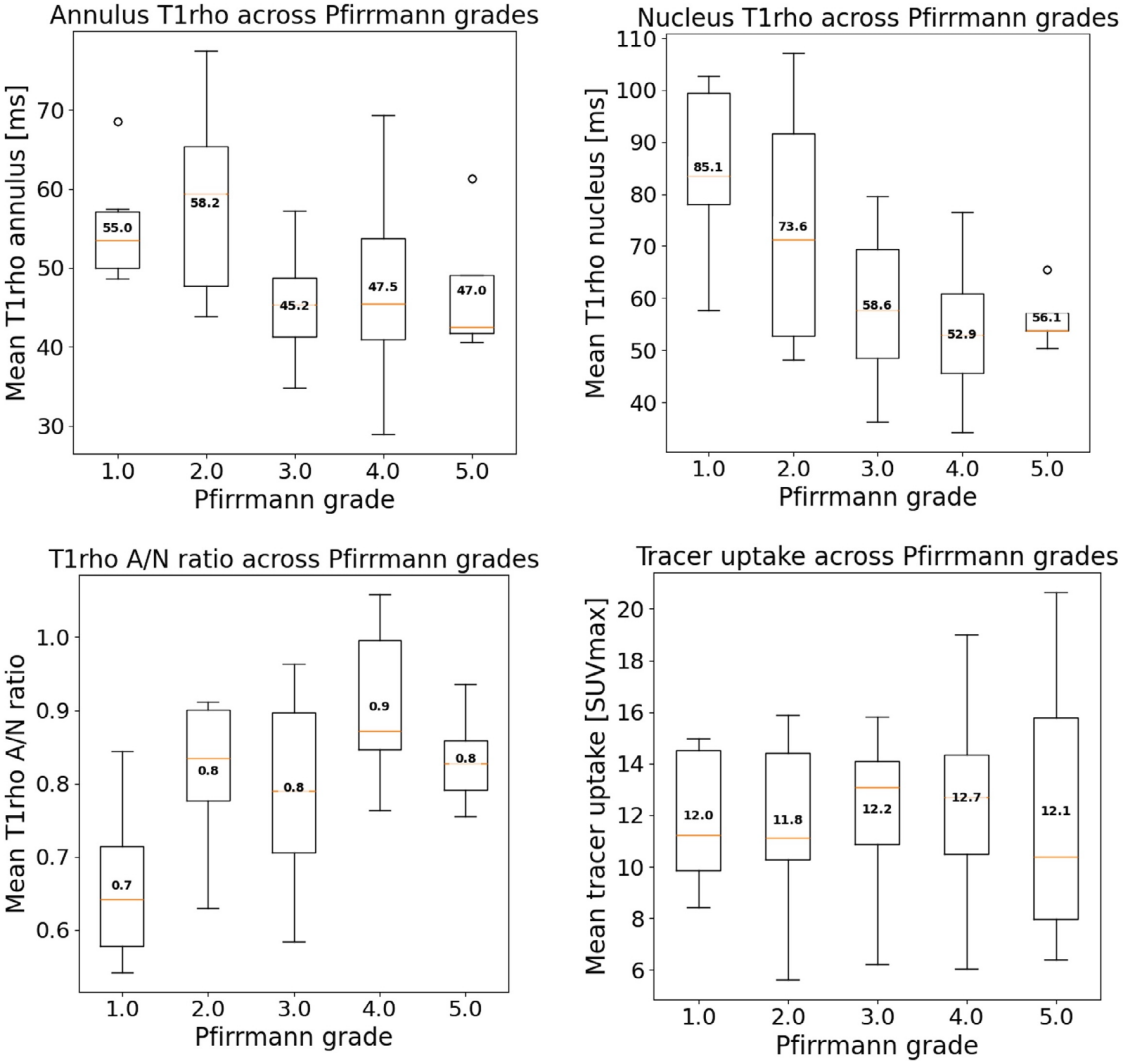
Quantitative metrics across Pfirrmann grades. A/N = annulus‐to‐nucleus ratio. Top left = annulus T1rho. Top right = nucleus T1rho. Bottom left = A/N ratio. Note different *y*‐axis scaling. Bottom right = Na[^18^F]F uptake (SUV_max_). Similar trends were observed in T2 data, which is not shown.

Cluster‐robust OLS regression (adjusted for age, sex, and BMI) revealed a significant association between Pfirrmann grade and nucleus T1rho (beta −6.93; 95% CI −12.49, −1.37; *p* = 0.015) and annulus‐to‐nucleus ratio (beta 0.04; 95% CI 0.01, 0.07; *p* = 0.010) but not annulus T1rho (beta −2.51; 95% CI −0.79, 16.05; *p* = 0.063). To visualize these associations, boxplots of quantitative dis metrics across Pfirrmann grades are provided in Figure 2. For T2 values, statistical significance for the association with Pfirrmann grades was missed: T2 nucleus (beta −4.78; 95% CI −10.54, −0.98; *p* = 0.104), T2 annulus (beta −2.37; 95% CI −4.77, −0.03; *p* = 0.053) and T2 annulus‐to‐nucleus ratio (beta 0.02; 95% CI −0.01, 0.04; *p* = 0.126). Endplate bone remodeling, quantified by Na[^18^F]F uptake narrowly missed statistically significant for association with Pfirrmann grades (beta 0.85; 95% CI −0.05, 1.75; *p* = 0.064).

### 3.3 Disco‐Vertebral Complex Lesions

Qualitatively assessed lesions of the disco‐vertebral complex, classified according to Modic, were overall rare, with 79% (68/86) of endplates showing no lesion. The most common lesion was Modic Type 2 (fat metaplasia), which was seen in 17% (15/86) of endplates, while only one Modic 1 lesion was identified. Average tracer uptake across endplates (SUV_max_) was 12.1 (±2.9); no clearly discernible trend regarding spatial distribution of uptake across the lumbar spine was observed. Mean values and observations per endplate are given in Table 2.

**TABLE 2:** Descriptive results for endplate metrics.

Endplate	Endplate lesions [%, *n*]				Na[^18^F]F uptake (SUV_max_) [mean, SD]
	None	Modic 1	Modic 2	Modic 3	
L1 inferior	100% (9/8)	0% (0/8)	0% (0/8)	0% (0/8)	12.0 (2.6)
L2 superior	88% (7/8)	0% (0/8)	12% (1/8)	0% (0/8)	12.2 (2.3)
L2 inferior	89% (8/9)	0% (0/9)	11% (1/9)	0% (0/9)	11.0 (2.9)
L3 superior	78% (7/9)	0% (0/9)	22% (2/9)	0% (0/9)	12.5 (3.7)
L3 inferior	78% (7/9)	0% (0/9)	11% (1/9)	11% (1/9)	13.0 (2.4)
L4 superior	78% (7/9)	0% (0/9)	11% (1/9)	11% (1/9)	12.1 (2.7)
L4 inferior	78% (7/9)	0% (0/9)	22% (2/9)	0% (0/9)	13.2 (3.7)
L5 superior	67% (6/9)	0% (0/9)	33% (3/9)	0% (0/9)	11.7 (3.5)
L5 inferior	63% (5/8)	12% (1/8)	25% (2/8)	0% (0/8)	11.5 (3.1)
S1 superior	75% (6/8)	0% (0/8)	25% (2/8)	0% (0/8)	12.1 (2.3)
Overall	79% (68/86)	1% (1/86)	17% (15/86)	2% (2/86)	12.1 (2.9)

Abbreviations: SD = standard deviation; SUV = standardized uptake value.

### 3.4 Association of Quantitative Disk and Endplate Metrics

**FIGURE 3:**
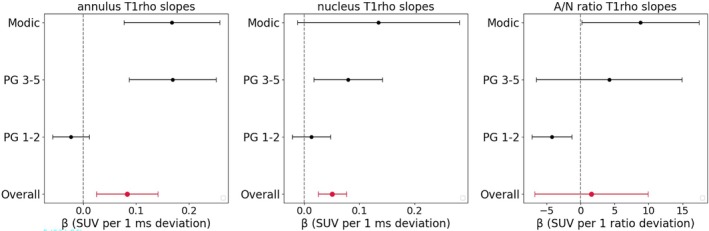
Forest plots for within‐spine associations (beta) between disk T1rho and Na[^18^F]F uptake. AN ratio = annulus‐nucleus ratio. PG = Pfirrmann grade. Effects (dots) with 95% CIs (whiskers). Main within‐spine effect shown in red, interactions shown in black. Vertical dotted line at zero. Similar trends without statistical significance were observed in T2 data, which is not shown.

To discern the association between quantitative disk metrics and endplate‐adjacent bone remodeling, we employed cluster‐robust OLS regression with Na[^18^F]F uptake as the dependent variable, and quantitative disk metrics, Pfirrmann grade, presence of Modic changes and annular fissure, sex, age, and BMI as predictors. Forest plots for the within‐spine slopes are provided in Figure 3. The strongest association with endplate‐adjacent bone remodeling, quantified by Na[^18^F]F uptake, was found for mean annulus T1rho—for this marker both within‐spine (beta 0.088; 95% CI 0.018–0.159; *p* = 0.014) and between‐spine (beta 0.229; 95% CI 0.084–0.374; *p* = 0.002) associations were positive and significant, showing that annulus hydration is positively associated with endplate‐adjacent bone turnover. For nucleus T1rho, only within‐spine associations were significant (beta 0.053; 95% CI 0.023–0.084; *p* = 0.001), and for A/N T1rho ratio neither within‐ nor between‐spine effects were significant (see overall effects in Figure 3, top row). There was a significant positive interaction with Modic changes (with steeper coupling in the presence of endplate changes) for annulus T1rho and A/N T1rho ratio, but not for nucleus T1rho (see Figure 3). Semiquantitative Pfirrmann grades added information independent of quantitative metrics (0.6–0.8 SUV per grade, *p* = 0.010–0.025) for annulus and nucleus T1rho, but not for the ratio between the two. Similar trends were found for quantitative T2 disk data, but as these results all missed statistical significance in this exploratory setting with limited statistical power, we decided to omit them from the results presentation for the sake of brevity and clarity.

### 3.5 Reproducibility of Measurements

Inter‐reader reliability measurements with a second radiologist (K.Z. 9 years of experience) were performed for both semiquantitative and quantitative measurements during precursor studies of this specific investigation [22, 26]. Interreader agreement (Cohen's kappa) for qualitative imaging assessments ranged between 0.66 (95% CI 0.42–0.84) and 0.67 (95% CI 0.38–0.95). Interreader agreement (intraclass correlation coefficient) for quantitative measurements was 0.98 (95% CI 0.98–0.99).

## 4 Discussion

Our exploratory study is the first to show the association of MR imaging biomarkers of disk degeneration and PET‐MRI detected bone remodeling at the vertebral endplates in a prospective cohort of LBP patients. Our main finding was a positive rather than the hypothesized negative ‐association between diskc hydration (particularly of the annulus fibrosus) and vertebral endplate bone remodeling. This unexpected direction points to a biological rather than purely mechanical coupling, possibly mediated by edematous changes in annular fissuring. The role of Modic changes as a modulator of this relationship further supports an inflammatory or fibrovascular component in this disk‐endplate crosstalk.

We originally hypothesized that dehydrated disks would lose their ability to effectively dissipate axial forces to the vertebral endplates, causing increased mechanical load and microfractures, repair of which would be quantifiable as increased bone remodeling on Na[^18^F]F PET‐MRI, resulting in negative associations between both markers. Assuming a largely mechanically driven process, we further hypothesized that nucleus hydration would be more closely negatively associated with these changes than annulus hydration. Our results were opposite of these findings, showing a small positive association between nucleus hydration and Na[^18^F]F uptake in the vertebral endplate (beta 0.051; *p* < 0.001), and a slightly stronger positive association for annulus hydration (beta 0.083, *p* = 0.005).

The positive associations we observed between disk hydration and endplate bone remodeling likely do not reflect healthy disk hydration, but rather edematous changes arising from annular tearing. T1rho values reflect interactions between extracellular matrix molecules and water, and are highest in the healthy disk, with particularly elevated values in the proteoglycan‐rich nucleus pulposus compared to the collagen‐rich annulus fibrosus [27]. In advancing disk degeneration, T1rho values decrease in both disk regions, but more steeply in the nucleus pulposus [28, 29]. Crucially, however, this relationship is altered in the presence of structural disk changes such as annular tearing: while Yang et al. found lower T1rho values in disks with tears [30], notably without controlling for overall disk degeneration, a study using quantitative T2 mapping, which is highly correlated with T1rho [30], conversely demonstrated increased values in the presence of annular tearing [31]. The positive associations in our data are therefore more consistent with this latter pattern, pointing to local edema rather than preserved hydration as the underlying signal. Advanced annular tearing and associated edema may be visualized as high‐intensity zones on T2‐weighted MRI, a finding that has in turn been associated with degenerative endplate changes including Modic changes [32]. Additionally, in advanced annular tearing, nuclear material may prolapse, which then may distort measurements that were intended as annular T1rho.

This interpretation is supported by the modulating role of Modic changes observed in our data: the association between annulus hydration and bone turnover was steeper in disk‐endplate units with Modic changes present, while nucleus hydration showed no such modulation. Given that prior work in this cohort demonstrated increased endplate bone remodeling in Modic 1 lesions [22], these findings together suggest an acceleration of bone turnover at the interface of edematous annular tissue and endplates already undergoing fibrovascular change [7], pointing to a biological rather than mechanical stimulus as the primary driver of endplate remodeling in this population.

We were able to replicate previous findings, showing a negative association between disk T1rho and Pfirrmann grades [28, 33], but the association only reached statistical significance for nucleus and annulus/nucleus ratio. Somewhat contrary to our initial hypothesis, endplates adjacent to disks with higher Pfirrmann grades did not show more bone remodeling‐the association only narrowly missed statistical significance, however (beta 0.85; 95% CI −0.05, 1.75; *p* = 0.064) and this result needs to be interpreted with caution considering the limited statistical power. In all models, Pfirrman grade retained an effect on endplate bone remodeling independent of quantitative markers of diskc health, however. This finding indicates that structural disk changes (i.e., height loss) may carry information significant to endplate biology beyond mere hydration status.

These findings carry potential clinical relevance for patient stratification in the context of endplate‐targeted therapies. Basivertebral nerve radiofrequency ablation has emerged as a treatment for vertebrogenic low back pain, with evidence supporting its efficacy specifically in patients selected by Type 1 or 2 Modic changes [34, 35]. Our data suggest that quantitative disk hydration markers ‐ particularly annular T1rho ‐ may provide complementary information beyond Modic grading alone in characterizing the disk‐endplate pathological unit. This is relevant given growing recognition that MRI phenotyping can help stratify LBP patients by underlying mechanism; a recent study demonstrated associations between specific MRI findings and inflammatory symptom profiles in nonspecific chronic LBP [36], underscoring the broader potential of imaging biomarker combinations to guide patient selection for mechanism‐targeted interventions.

The main limitations of our study arise from the small sample size of only nine subjects and thus limited statistical power in our study sample. Our post hoc power analysis showed a slightly lower power than conventionally required (73%), so it needs to be cautioned that true effects may have been missed. In this modestly sized study population, we were furthermore unable specifically to investigate sex interactions, despite mounting evidence of their importance in degenerative musculoskeletal conditions. This also prompted us to not include quantitative disk T2 data, which showed similar trends to the ones seen for T1rho but missed statistical significance in the main analysis. Within this exploratory study, we also did not have a broad representation of different grades and types of degeneration of the disco‐vertebral complex, seen most clearly in the small number of degenerative Modic changes observed in our sample. Relatedly, Modic changes in our sample were predominantly Type 2, with only a single Type 1 lesion identified; as MC types are thought to reflect distinct stages of a pathobiological continuum (with Type 2 representing a more quiescent fatty end stage), the modulating effect of Modic changes on the disk‐endplate coupling observed here likely reflects Type 2 biology, and it remains an open question whether a cohort enriched for Type 1 changes would show similar results. Furthermore, our cohort represented predominantly early to intermediate disk degeneration (median Pfirrmann Grade 3); it is possible that the mechanically driven endplate remodeling we originally hypothesized would only become apparent at more advanced degeneration stages and that the biological coupling we observed reflects pathways dominant in earlier disease. We also did not have data on axial loading conditions of our subjects, which may have introduced bias from unmeasured effects, as physical activity is linked to disk hydration [37] and has also been connected to bone remodeling in osteoarthritis [38, 39].

Future research should focus on refining and improving quantification of Na[^18^F]F uptake, for example by utilizing automated pipelines with (preferably volumetric) segmentation of vertebral endplates, which would allow for a more detailed analysis, for example, distinguishing between central and peripheral as well as superficial and deep regions of the vertebral endplate. Furthermore, a broader variety of uptake markers, including SUV_mean_ and SUV_peak_ could be integrated in future analyzes. The work could also be expanded to include shape or volume analyzes of vertebral disks, to attempt a quantification of the effects seen in the retained explanatory power of Pfirrmann grades in this dataset. Care should be taken to integrate qualitative assessments such as annular tearing future analytic frameworks, to facilitate a comprehensive interpretation of T1rho and other quantitative disk values.

In conclusion, our study provides evidence of a positive association between hydration status of the annulus fibrosus and bone remodeling of the vertebral endplates in individuals with LBP. These findings support claims to a biological rather than mechanical stimulus for increased bone remodeling in vertebral endplate degeneration, possibly triggered by annular fissuring and subsequent edema and local tissue response.

## Author Contributions

V.K. and S.M. conceptualized the study. V.K., C.T.C., and S.M. developed the methodology. M.H. and R.B. contributed software. V.K., K.Z., M.H., I.R., and E.B. performed data curation. V.K. and K.Z. conducted the investigation. K.Z. performed validation. V.K., K.Z., M.H., and R.B. carried out the formal analysis. C.T.C. and S.M. supervised the project and acquired funding. V.K. and K.Z. contributed to visualization. I.R. and E.B. administered the project. R.B. and S.M. provided resources. V.K. drafted the original manuscript. V.K., K.Z., M.H., C.T.C., R.B., I.R., E.B., and S.M. reviewed and edited the manuscript. All authors approved the final version of the manuscript.

## Funding

This work was supported by the National Institutes of Health (UH3AR076724).

## Conflicts of Interest

The authors declare no conflicts of interest.

## Data Availability

The data that support the findings of this study are available on request from the corresponding author. The data are not publicly available due to privacy or ethical restrictions.
